# Historical Introgressions from a Wild Relative of Modern Cassava Improved Important Traits and May Be Under Balancing Selection

**DOI:** 10.1534/genetics.119.302757

**Published:** 2019-10-17

**Authors:** Marnin D. Wolfe, Guillaume J. Bauchet, Ariel W. Chan, Roberto Lozano, Punna Ramu, Chiedozie Egesi, Robert Kawuki, Peter Kulakow, Ismail Rabbi, Jean-Luc Jannink

**Affiliations:** * Section on Plant Breeding and Genetics, School of Integrative Plant Sciences, Cornell University, Ithaca, New York 14850; † Boyce Thompson Institute, Ithaca, New York 14853; ‡ Institute for Genomic Diversity, Cornell University, Ithaca, New York 14850; § International Programs, College of Agriculture and Life Sciences, Cornell University, Ithaca, New York 14850; ** National Root Crops Research Institute (NRCRI), Umudike, Umuahia, 440221, Nigeria; †† International Institute of Tropical Agriculture (IITA), Ibadan 200001, Nigeria; ‡‡ National Root Crops Resources Research Institute, Namulonge, Uganda; §§ United States Department of Agriculture – Agriculture Research Service, Ithaca, New York 14850

**Keywords:** cassava, introgression, genetic architecture, heterozygote advantage, balancing selection, heritability

## Abstract

Introgression of alleles from wild relatives has often been adaptive in plant breeding. However, the significance of historical hybridization events in modern breeding is often not clear. Cassava (*Manihot esculenta*) is among the most important staple foods in the world, sustaining hundreds of millions of people in the tropics, especially in sub-Saharan Africa. Widespread genotyping makes cassava a model for clonally propagated root and tuber crops in the developing world, and provides an opportunity to study the modern benefits and consequences of historical introgression. We detected large introgressed *Manihot glaziovii* genome-segments in a collection of 2742 modern cassava landraces and elite germplasm, the legacy of a 1930s era breeding to combat disease epidemics. African landraces and improved varieties were, on average, 3.8% (max 13.6%) introgressed. Introgressions accounted for a significant (mean 20%, max 56%) portion of the heritability of tested traits. *M. glaziovii* alleles on the distal 10 Mb of chr. 1 increased dry matter and root number. On chr. 4, introgressions in a 20 Mb region improved harvest index and brown streak disease tolerance. We observed the introgression frequency on chr. 1 double over three cycles of selection, and that later stage trials selectively excluded homozygotes from consideration as varieties. This indicates a heterozygous advantage of introgressions. However, we also found that maintaining large recombination-suppressed introgressions in the heterozygous state allowed the accumulation of deleterious mutations. We conclude that targeted recombination of introgressions would increase the efficiency of cassava breeding by allowing simultaneous fixation of beneficial alleles and purging of genetic load.

INTERSPECIFIC hybridization has provided an important source of adaptive genetic variation during evolution in many organisms including humans (Harris and Nielsen 2016; Juric *et al.* 2016), cattle (Wu *et al.* 2018) and maize (Hufford *et al.* 2013). Indeed introgression between many crops and their undomesticated relatives has occurred in both directions Ellstrand *et al.* (2013), naturally in farmers fields and deliberately by plant breeders (Jarvis and Hodgkin 1999; Zamir 2001; Hajjar and Hodgkin 2007; Warschefsky *et al.* 2014). Introgression can have serious population genetic consequences including genomic inversions and other structural variations, suppression of recombination and segregation distortion, inbreeding depression, and hybrid sterility (Haldane 1957; Rieseberg *et al.* 2000; Fijarczyk and Babik 2015). Despite many individual examples, the consequences of historical introgressions, both positive and negative, especially at the quantitative genetic level, is rarely simultaneously understood.

Cassava (*Manihot esculenta*) is among the most important staple foods in the world, sustaining hundreds of millions of people in the tropics, especially in sub-Saharan Africa (http://faostat.fao.org). Cassava is a clonally propagated staple food crop, grown throughout the tropics for its starchy storage roots. In recent years, cassava has emerged from orphan-crop status as a model for plant breeding in the developing world, especially among outbreeding noncereals and vegetatively propagated root and tuber crops (Ceballos *et al.* 2012; Prochnik *et al.* 2012; International Cassava Genetic Map Consortium (ICGMC) 2014; Wolfe *et al.* 2017).

The history of cassava breeding includes periodic tapping of wild congeneric relatives as sources of useful genetic variation (Jennings and Iglesias 2001; Hajjar and Hodgkin 2007). In the early twentieth century, cassava production in Africa faced a grave threat in the form of mosaic disease, caused by an insect-vectored gemini virus. Records indicate that an initial worldwide search for resistant cultivated cassava was conducted (Storey and Nichols 1938; Nichols 1947; Jennings 1957; Fauquet 1990). Failing to find native resistance, breeders at the Amani research station in Tanzania introgressed resistance from the Ceara rubber tree (*Manihot glaziovii* Muell. Arg.) (Storey and Nichols 1938; Nichols 1947; Jennings 1957; Hahn *et al.* 1980b; Fauquet 1990).

Three backcrosses of hybrids to *M. esculenta* produced acceptable levels of resistance and storage root yield (Jennings 1957; Hahn *et al.* 1980b), leading to the distribution of mosaic-tolerant varieties to farmers in the local area of Amani (Jennings 1957; Legg and Thresh 2000), and the eventual end of the first mosaic disease epidemics by the 1940s (Jennings 1957; Legg and Thresh 2000). Descendants of these original hybrids became key founders of modern breeding germplasm (Ekandem 1970; Hahn *et al.* 1980b; Fauquet 1990). The Amani-derived lines have been identified as important sources of resistance against cassava mosaic disease (CMD) (Fregene *et al.* 2000; Lokko *et al.* 2006), brown streak disease (CBSD) (Hillocks and Jennings 2003), and bacterial blight (Hahn *et al.* 1980a).

Large genome-segments derived from *M. glaziovii* were recently discovered in a sample of African genotypes, suggesting that historical introgressions remain important today (Bredeson *et al.* 2016). Several other studies have identified quantitative trait loci (QTL) in these regions, leading us to hypothesize that *M. glaziovii* alleles confer CBSD resistance (Bredeson *et al.* 2016; Nzuki *et al.* 2017; Kayondo *et al.* 2018), and possibly increased storage root dry matter content (Rabbi *et al.* 2017).

Widespread genotyping for genomic selection (GS) in African cassava breeding (http://www.nextgencassava.org) makes cassava a model for root and tuber crops in the developing world, and provides an opportunity to study the modern benefits and consequences of historical introgression. We leveraged publicly available data (http://www.cassavabase.org) from >2742 breeding lines, land races, and local varieties, with both field phenotypes and genome-wide marker records (Wolfe *et al.* 2017) as well as whole-genome sequences (WGS) (Ramu *et al.* 2017). First, we investigated the legacy of *M. glaziovii* introgression by determining its extent in the germplasm and the associated population structure. We employed a combination of genetic variance partitioning, genome-wide association analysis and genomic prediction to quantify the location, effects and overall importance of introgressed alleles for key cassava traits, and, thus, for cassava breeding. Finally, we study three generations of genomic selection progenies to understand the role of introgressions in modern cassava breeding.

## Methods

### GBS and WGS datasets

The HapMapII dataset is a collection of 30× WGS, which has been previously described (Ramu *et al.* 2017). HapMapII contains 238 accessions including 8 *M. glaziovii*, 11 *M. esculenta* x *M. glaziovii* hybrids, 16 *M. flabellifolia* (wild progenitor of cassava), a few other wild relatives, and 200 cultivated *M. esculenta* samples genotyped at ∼28 M SNPs. HapMapII was the basis for identifying introgression diagnostic markers (IDMs), which could be used to detect *M. glaziovii* introgressions in the genome of additional samples. The rest of the germplasms we analyzed were genotyped using the genotyping-by-sequencing (GBS) approach (Elshire *et al.* 2011; Hamblin and Rabbi 2014). The overall GBS pipeline we employed for quality control and genotype imputation have been described previously (Hamblin and Rabbi 2014; Wolfe *et al.* 2017; Ozimati *et al.* 2018).

We have included GBS genotypes and phenotypes from three cassava breeding programs: National Root Crops Research Institute (NRCRI, Umudike, Nigeria), International Institute of Tropical Agriculture (IITA, Ibadan, Nigeria), and National Crops Resources Research Institute (NaCRRI, Namulonge, Uganda).

The genomic selection (GS) training populations for the three programs, IITA, NRCRI (NR), and NaCRRI (UG), have all been previously described (Ly *et al.* 2013; Wolfe *et al.* 2016a,b, 2017; Okeke *et al.* 2017; Rabbi *et al.* 2017; Elias *et al.* 2018; Kayondo *et al.* 2018; Ozimati *et al.* 2018). Additional samples were sourced from IITA’s Genetic Gain (GG) (Okechukwu and Dixon 2008) and Local Germplasm (LG) populations. The LG is a collection of landraces and local varieties originating mostly, but not exclusively, in west Africa. IITA also contributed GBS data from a panel of Latin American accessions obtained through its collaboration with the International Center for Tropical Agriculture (CIAT). Finally, we included GBS data from three consecutive IITA progeny generations (herein C1, C2, and C3), with C1 being descended from selected GG parents, C2 being descended from crosses among selected C1 parents, and so on (Wolfe *et al.* 2017).

Except for the CIAT collection, our GBS samples were derived from the following pipeline: a reference panel of 4629 accessions, consisting of all African germplasm available that were not classified as GS progenies (*i.e.*, three training populations plus assorted landraces) was assembled. Sites were removed if >80% had zero reads. Imputation was done with Beagle v4.0. The imputed data were filtered, keeping sites with allelic R-square (INFO/AR2 field of the VCF) ≥0.3. The imputed and filtered reference set was then used to impute the remaining IITA GS progeny.

The CIAT lines were part of a separate study and thus were processed and imputed along with additional samples (not published). For those data, before imputation, genotype calls were allowed only if a minimum of one read and a maximum of 50 reads were present for each individual at each site. We removed loci with >80% missing and an average mean depth >120. We also thinned markers within 5 bp of each other. These quality control procedures were implemented using the VCFtools (v0.1.14) software package (Danecek *et al.* 2011). We then imputed missing data using Beagle (v4.1) using the *gl* mode, *window* = 2250, *overlap* = 225, and *niterations* = 10 (Browning and Browning 2007, 2016). Postimputation, we removed markers based on the allelic *R*^2^ (AR2 < 0.3) (Browning and Browning 2009).

### Introgression diagnostic markers

We used an ancestry informative marker approach, similar to that of Bredeson *et al.* (2016) in order to detect introgressed *M. glaziovii* genome segments. Our approach relies on the comparison of a set of pure (*i.e.*, nonintrogressed) *M. esculenta* (Me reference) to a collection of *M. glaziovii* (Mg reference). From comparison of these two reference panels, we identified introgression diagnostic markers (IDM) that can be used for detecting *M. glaziovii* segments in an admixed sample. We used two criteria to classify a single nucleotide polymorphism (SNP) as an introgression diagnostic. Either the SNP is fixed for different alleles in the two (Me and Mg) reference panels (“Strict” IDMs), or the SNP must be fixed in the Me reference but polymorphic in the Mg sample (“GlazPoly” IDMs). The rationale for “GlazPoly” IDMs is that, if we identify alleles that are *only* present in the *M. glaziovii* panel and *not* in the pure *M. esculenta*, then finding the *M. glaziovii* alleles at those sites in an introgressed individual would be diagnostic, or at least contribute to our confidence, that an *M. glaziovii* genome segment was present.

For our *M. glaziovii* reference panel, we selected seven samples (GLA59008, GLA59008-1, MAN00401, MGLAZIOVII, Mglaziovii, MglazioviiR, MglazioviiS) that were marked as *M. glaziovii* in HapMapII (Table S1 of Ramu *et al.* (2017) and included one additional sample (IRWA02712), because in admixture and clustering analyses (not shown) IRWA02712, though it was marked as *M. irwinii*, it was indistinguishable from *M. glaziovii*.

The first step toward identifying a set of IDMs was to define panels of *M. glaziovii* and *M. esculenta*. HapMapII includes 16 samples of the wild progenitor of cassava, *M. flabellifolia*. We exclude these samples from our study.

Defining a reference panel of “pure” *M. esculenta* required greater care than for *M. glaziovii* since we know our sample potentially includes admixed individuals but do not yet know which. To start, we excluded the hybrid, *M. flabellifolia* and wild relative samples from consideration. In Bredeson *et al.* (2016), an analysis using the software frappe (Tang *et al.* 2005) was the primary basis for defining “pure” cassava. In order to ensure that, on a segment-to-segment basis, our cassava reference set truly did not contain *M. glaziovii* segments, we defined Me reference panels on a per-window basis. For every 1000 SNPs, we calculated the pairwise Hamming distance [as implemented by the –distance flag in plink1.9beta3.3, http://www.cog-genomics.org/plink/1.9/, Chang *et al.* (2015)] between all HapMapII samples. To make this procedure more computationally tractable, we first linkage disequilibrium (LD) pruned the 28 M SNP set using the plink1.9 –indep-pairwise flag with a window size of 50 SNPs, step size of 10, and an LD $r^{2}$ threshold of 0.3. After LD-pruning, the HapMapII dataset had 4,952,655 SNPs left. For each 1000 SNP window, we calculated the mean Hamming distance between the Mg reference panel and each cultivated cassava sample (MeanGlazDist for short).

Before making decisions about which cassava clones were genetically distant enough from *M. glaziovii* to use as a window-specific reference panel, we plotted summary statistics (Supplemental Material, Figure S1). Specifically, we calculated the mean, median, maximum, and SD of MeanGlazDist variable for each window (Figure S2). We noted some outlier windows at the end of chromosomes, where all summary statistics are very close to zero. We exclude these windows from further consideration by filtering cases with MeanGlazDist <200. We then select as Me reference for each window, the 10 clones with greatest MeanGlazDist in that window.

In order to apply IDMs for detecting introgressions in the GBS data, we restrict our downstream analyses to the 149,098 GBS sites intersecting both GBS and WGS datasets.

### Principal components analysis

We conducted a principal components analysis (PCA) on three marker sets: all markers (IDM+non-IDM), IDM markers, and non-IDM markers. For this analysis, non-IDM markers that were later determined to be in strong LD with the set of IDM (see “*Introgression tagging variants*” below), were included only in the “all markers” analysis. Only the populations UG, GG, NR, and LG (*N* = 2742) and SNPs with minor allele frequency (MAF) >0.01 were included in the PCAs. PCA was conducted using the *prcomp()* R function, with center and scale arguments set as TRUE.

The PCA and many other downstream analyses were conducted on allelic dosage matrices, where the genotypes for each individual (rows) and each SNP (columns) is represented as 0, 1, 2. Note that our dataset contains noninteger values between 0 and 2, representing the uncertainty of imputation. For the non-IDM and tag-IDM, the counted allele is the default for the *plink* “*–recode*” function. We coded the IDM dosages so the counted allele is the *M. glaziovii* diagnostic allele (*plink* “–recode-allele”). Doing this enables us to interpret eigenvector coefficients (PC loadings) *and* SNP effects [see section on genome-wide association analysis (GWAS) below] for those markers relative to introgressions. For example, a positive loading for an IDM SNP means individuals at the high end of that principal component are more likely to have a *M. glaziovii* diagnostic allele at that site than individuals at the low end.

### Mapping introgressions in windows

GBS (Elshire *et al.* 2011; Hamblin and Rabbi 2014) produces genotype data with a high proportion of missing sites and a low average read depth, which necessitates imputation (Browning and Browning 2007; Chan *et al.* 2016) for most applications (see above). Individual IDM genotype calls may be incorrect. This means the use of IDM to detect introgressions have some probability of both false positives and negatives. One step we took to reduce the potential noise from individual IDMs was to follow a window-based approach similar to Bredeson *et al.* (2016). We computed the average *M. glaziovii* dosage across IDMs in 250 kb, nonoverlapping windows across the genome (referred to also as *DoseGlaz)*. The window-based dosages were used to generate genome-wide maps of introgression status for each sample and for GWAS (see below).

### Interpolating marker genetic distances

Marker genetic distances were interpolated using the consensus genetic map from the International Cassava Genetic Map Consortium (ICGMC) (2014) based on the cassava reference genome v4 (Prochnik *et al.* 2012). The 22,404 ICGMC Markers flanking sequences (105 bp) were filtered on biallelic variation, and subjected to a nucleotide BLASTN procedure (Camacho *et al.* 2009) against the cassava reference genome version 6 (Bredeson *et al.* 2016) to obtain their new physical positions. Using a custom python script and these inferred ICGMC v6 positions, we interpolated all GBS markers in our data.

### Comparing introgressions among populations

There were eight data sets (GG, LG, NR, UG, C1, C2, C3, and CIAT) for which we wanted to compare introgression status. We computed both the *M. glaziovii* allele frequency and homozygosity rate. For each population, these summaries were made both on a per IDM and a per individual basis. Further, we examined differences between populations on a genome-wide basis as well as in focal introgression regions on chr. 1 (from 25 Mb+) and chr. 4 (5–25 Mb).

### HAPMIX in HapMapII

In order to provide additional confidence in the introgressions we detect using IDMs, we applied an alternative approach to the HapMapII dataset. We used HAPMIX (Price *et al.* 2009)—a haplotype-based method for local ancestry inference in populations formed by two-way admixture to estimate a probability of introgression at each site. HAPMIX assumes that the admixed population of interest arose from a single admixture event between two ancestral populations. In our case, the two ancestral populations are *M. glaziovii* and *M. esculenta*. Before running HAPMIX, we used HapCUT (Bansal and Bafna 2008) on already Beagle-imputed and phased HapMapII dataset in order to improve local phasing.

#### Defining the two reference populations and the admixed sample:

We needed two reference populations: reference population 1, consisting of individuals related to the true *M. glaziovii* ancestral population, and reference population 2, consisting of individuals related to the true *M. esculenta* ancestral population. We used the same eight *M. glaziovii* individuals that were used to define IDMs. To define reference population 2, we selected the 10 *M. esculenta* individuals in HapMapII (out of the 217 *M. esculenta*) that were most genetically distant from the eight *M. glaziovii* samples based on IDMs analysis. To define them, we simply summed the *MeanGlazDist* values across each individual’s genome and selected the top 10. They were: CW45617, CPCR15B91, CPCR27B7, CPCR27B17, MBRA685, UG08S0P003, CR4442, CPCR24B3, BRA8565, and UG08S0P005. The remaining 207 *M. esculenta* served as our admixed sample.

#### Specification of HAPMIX parameters:

HAPMIX requires specification of nine model parameters: (1) the average number of generations since admixture, *T*; (2–3) the rates at which there is copying of ancestry segments from the “wrong” population, $p_{1}$ and $p_{2}$; (4–6) “mutation” parameters $\theta_{1}$, $\theta_{2}$, and $\theta_{3}$; (7) the probability that a given segment of an admixed haplotype originates from ancestry population 1 and population 2, $\mu_{1}$ and $\mu_{2}$; and (8–9) the “recombination” rate between haplotypes within reference population 1 and 2, $\rho_{1}$ and $\rho_{2}$. We selected parameter values via a process of trial and error, running HAPMIX on the 11 *M. esculenta* x *M. glaziovii* hybrids in HapMapII (results not shown). For each hybrid, when tuned properly, HAPMIX should infer the presence of one *M. glaziovii* allele at each site (or at least, a large proportion of sites). We selected the following parameter settings for HAPMIX: T=4, $p_{1}=0.05$, $p_{2}=0.05$, $\theta_{1}=0.2$, $\theta_{2}=0.2$, $\theta_{3}=0.01$, $\mu_{1}=0.2$ (and $\mu_{2}=1-\mu_{1}$), $\rho_{1}=700$, and $\rho_{2}=900$. We ran HAPMIX using the HAPMIX_MODE=“DIPLOID”, OUTPUT_DETAIL=“HAPLOID_FILES”, and THRESHOLD = 0.9.

### Introgression tagging variants

Our next major objective was to quantify the phenotypic impact of segregating *M. glaziovii* genome segments on the germplasm. We used genomic mixed models to estimate the amount of genetic variance attributable to introgression regions relative to the rest-of-the-genome (de los Campos *et al.* 2015; Speed and Balding 2015; Pino Del Carpio *et al.* 2018). If unaddressed, LD between IDM and non-IDM SNP sets will lead to nonindependent estimates of genetic variance (Speed *et al.* 2012; Bulik-Sullivan *et al.* 2015; Finucane *et al.* 2015; Pino Del Carpio *et al.* 2018). The variance arising from introgression regions might then be captured by the nonintrogression regions and vice versa.

It is impossible to entirely eliminate this problem: long distance LD exists in populations of clones driven by population structure and familial relatedness. However, to reduce nonindependence, for every SNP not previously identified as IDM, we calculated two statistics. Both are based on pairwise LD $r_{\text{LD}}^{2}$ statistics as computed in R as the squared correlation among allelic dosages. From these $r_{\mathrm{LD}}^{2}$ values, we first determined the maximum LD (maxLD) observed between each non-IDM SNP and the set of IDMs. Our rationale here was that non-IDM SNPs in very high LD with even one IDM SNP could explain genetic variance attributable to the same causal variants. Second, we calculated the total LD (totalLD) between each non-IDM and the entire set of IDMs. This metric is essentially the same as the LDscore, which is calculated on a window-basis (Speed *et al.* 2012; Bulik-Sullivan *et al.* 2015; Finucane *et al.* 2015; Pino Del Carpio *et al.* 2018). The totalLD, we reasoned, might provide an even better idea (compared to maxLD) of the degree to which an non-IDM SNP “tagged” the introgressed regions of the genome.

We needed an at least semi-objective approach to choose a threshold for declaring SNPs as “tagging” IDMs or not. We tested a range of LDscore (300–1500, interval 200) and maxLD (0.1–1, interval 0.2). For each threshold, we partitioned the SNPs into IDM, non-IDM, and tag-IDM, accordingly. We then constructed four kinship matrices using the *A.mat* function from the rrBLUP R package (Endelman 2011): tag-IDM, IDM, non-IDM, and IDM+tag-IDM. We used the correlations between the upper off-diagonals as a proxy for the independence of genetic variance components that might result. Our objective was therefore to partition the SNPs using LDscore and/or maxLD such that we maximize the cor(tag-IDM, IDM) and minimize both cor(tag-IDM, non-IDM) and cor(IDM+tag-IDM, non-IDM). The correlation between the kinships using the original partition of IDM and non-IDM (0.37) was the baseline to improve upon.

We chose to use a LDscore threshold of 500, because they were more similar in the kinship they measured to the IDM than to the non-IDM (Figures S5–S7 and Tables S1 and S4). By redesignating these originally non-IDM SNP as tag-IDM and including them in the kinship matrix with IDMs, we reduced the correlation of IDM and non-IDM kinships to 0.30. We included tag-IDM in the IDM kinship matrices used in all subsequent analyses. With this procedure, we hoped to improve our ability to distinguish introgression-associated (IDM + tag-IDM) from the rest of the genetic variance (non-IDM) in key cassava traits.

### Field trials

#### Trials chosen:

For this study, we compiled data from 68 field trials (42 IITA, 5 NaCRRI, 21 NRCRI), which were scored for nine traits. NaCRRI, NRCRI, and IITA (see section *Datasets* above) have GS programs as part of a project called Next Generation Cassava Breeding (www.nextgencassava.org). For the NR and UG populations, the trials included in our analyses comprise the GS training populations (TPs). For IITA, trials from both the GG (original TP) and LG (landrace and local germplasm) populations were included. There were a total of 2742 phenotyped clones in the dataset.

With the exception of the LG dataset, versions of most of these data have been analyzed in other publications (Wolfe *et al.* 2016a,b, 2017; Rabbi *et al.* 2017; Kayondo *et al.* 2018). Versions of these trials are available from the online database www.cassavabase.org. In addition, we summarize the trials in terms of the number of observations (Nobs), clones (Nclone), reps (Nrep), and the ratio of Nobs/Nclone (ObsToCloneRatio) per-Institute-per-Trial (Table S6).

#### Traits scored:

Cassava faces a number of pest and disease problems in Africa (Legg *et al.* 2014). We analyzed severity scores, which are on a standard scale of 1 (no symptoms) to 5 (very severe symptoms), for three diseases: cassava brown streak disease root necrosis (CBSDRS) and foliar (MCBSDS), season-wide mean cassava mosaic disease (MCMDS), and cassava bacterial blight (MCBBS).

We also scored five yield-related traits: dry matter content (DM), fresh root weight (RTWT), fresh shoot weight (SHTWT), root number per plot (RTNO), and harvest index (HI). MCMDS and MCBSDS are the mean of measurements taken at up to three time points throughout the season: 1, 3, and 6 months after planting (MAP). Dry matter content is the percentage of dry root weight relative to fresh root weight (RTWT). At IITA, DM was measured by drying 100 g of fresh roots in an oven whereas at NRCRI and NaCRRI, the specific gravity method (Kawano *et al.* 1987) was used. Both RTWT and SHTWT were expressed in kilograms per plot. The HI was the ratio of RTWT to RTWT plus SHTWT. RTNO was the number of roots harvested from each plot. For all analyses below, RTNO, RTWT, and SHTWT were natural-log transformed to improve homoscedasticity of residuals.

Refer to the Cassava Trait Ontology (http://www.cropontology.org/ontology/CO_334) and our previous publications for additional details (*e.g.*, Wolfe *et al.* (2017) and Ozimati *et al.* (2018)).

There were as many as 68 trials scored for MCMDS, and as few as five for CBSDRS/MCBSDS (NaCRRI only) (Table S6).

### Genetic variance from introgressions

We fit linear mixed-models to the field trial data described above (Henderson 1975; Gianola and Rosa 2015). The genotypic effect was modeled as random, with the covariances among levels assumed as proportional to a known coancestry coefficient. The variance component associated with this term in the model is an estimate of the additive genetic variance (allowing us to compute the heritability, $h^{2}$) (Yang *et al.* 2010; de los Campos *et al.* 2015; Pino Del Carpio *et al.* 2018). We started with the basic mixed model of form described above:

y=Xβ+Zg+ϵ

In this model, *y* is a n(observations)x1 vector of phenotypic records, *X* is the *n* x *p* design matrix relating observations to corresponding *p* levels of the fixed-effects. The *p* x 1 vector *β* contains the fixed-effect estimates. The *n* x *q* design matrix *Z* related the records in *y* to the *q* levels of the random effects vector *g*, in this case *g* is the genotype (unique cassava clone) effect. The nx1 random vector *ϵ* is the residual or error term.

We assume the following about the random effects vector:

$$\left(\begin{array}{l} g\\ \epsilon \end{array}\right)∼N\left(0,\left[\begin{array}{cc} \sigma_{g}^{2}K_{g} & 0\\ 0 & \sigma_{\epsilon }^{2}I \end{array}\right]\right)$$

Both random effects have a mean of 0. The genomic relationship matrix $K_{g}$ is a square symmetric covariance matrix, which we constructed using genome-wide SNP markers and the first method from VanRaden (2008) as implemented in the *A.mat()* function in the *rrBLUP* R package (Endelman 2011). *I* is the identity matrix, which specifies the usual independent and identically distributed constraint on the residuals. Given $K_{g}$, $\sigma_{g}^{2}$ is an estimate of the additive genetic variance, and *g* (the best linear unbiased predictors, BLUPs) are, in this case, often called genomic-estimated breeding values (GEBVs).

We want to partition the total heritability $\left(h_{g}^{2}\right)$ into a component due to the regions with introgressed *M. glaziovii* alleles $\left(h_{\text{IDM}}^{2}\right)$ and a component attributed to regions without introgressions $\left(h_{\text{nonIDM}}^{2}\right)$. We do this by constructing two GRMs, one with markers from IDMs plus tag-IDMs $\left(K_{\text{IDM}}\right)$ and the other with the rest of the markers $\left(K_{\text{nonIDM}}\right)$. We fit the following, expansion on the mixed-model above:

$$y=X\beta +Zg_{\text{IDM}}+Zg_{\text{nonIDM}}+\epsilon$$

$$\left(\begin{array}{c} g_{\text{IDM}}\\ g_{\text{nonIDM}}\\ \epsilon \end{array}\right)∼N\left(0,\left[\begin{array}{ccc} \sigma_{\text{IDM}}^{2}K_{\text{IDM}} & 0 & 0\\ 0 & \sigma_{\text{nonIDM}}^{2}K_{\text{nonIDM}} & 0\\ 0 & 0 & \sigma_{\epsilon }^{2}I \end{array}\right]\right)$$

Here, *Z* is the design matrix for both $g_{\text{IDM}}$ and $g_{\text{nonIDM}},$ as long as the order of the rows/columns of the two corresponding kinship matrices is the same.

For simplicity, we will sometimes refer to the two models described above as the ALL and the PARTITIONED models, respectively.

#### Per-trial analysis:

Our first analysis was on a per-trial basis, where a “trial” is defined as a unique experiment planted in a single location-year.

In addition to the two genetic models described above, for each trial, we fit three additional models:

IID:

$$y=X\beta +Zg_{\text{IID}}+\epsilon$$

$$g_{\text{IID}}∼N\left(0,\sigma_{\text{IID}}^{2}I\right)$$

IDM:

$$y=X\beta +Zg_{\text{IDM}}+\epsilon$$

$$g_{\text{IDM}}∼N\left(0,\sigma_{\text{IDM}}^{2}K_{\text{IDM}}\right)$$

IDMnull:

$$y=X\beta +Zg_{\text{nonIDM}}+\epsilon$$

$$g_{\text{nonIDM}}∼N\left(0,\sigma_{\text{nonIDM}}^{2}K_{\text{nonIDM}}\right)$$

We also fit a NULL, with no genetic component. In some cases, for the NULL model, when no nongenetic effects were relevant, we fit an intercept, or an intercept + NOHAV fixed-effects model, using the *lm* function in R (v3.4.3). For the rest of the models described above, we fit them with the *mmer* mixed-model solver function in the *sommer* (v3.1) R package (Ly *et al.* 2013; Covarrubias-Pazaran 2016).

Since a trial is a single location-year, the only fixed-effect was the number of plant stands harvested (NOHAV), fit for RTWT, RTNO, and SHTWT (Ly *et al.* 2013). Nongenetic random effects were added, where relevant, on a trial and breeding program-specific basis. Replication effects were fit to replicated trials for all three breeding programs. For NRCRI and NaCRRI, two random effects, one for complete blocks (replication effects) and another for incomplete blocks nested within replications. All nongenetic components were assumed to be *i.i.d*. (covariance equal to *I*).

For model comparisons, described below, we manually calculated the Akaike Information Criterion (AIC) as AIC=2*npar−2*log Lik, where npar is the number of fixed+random parameters fitted, and logLik is the log-likelihood from *sommer*’s solution.

In the case of nested model comparisons described below, we conducted likelihood ratio tests (LRT) to determine the significance of individual random effects. Using the likelihoods from the models describe above, we compared $2*\left(logLik_{\text{full}}-logLik_{\text{null}}\right)$ to a chi-square distribution with $df=npar_{\text{full}}-npar_{\text{null}}$. Here the *null* model refers to a model “nested” within the *full* model, *i.e.*, with one or more random-effect dropped.

Each genetic model was compared to the NULL (nongenetic) model $\left(LRT_{\text{null}}\right)$.

#### Multi-trial analysis:

Using the results of the trial-by-trial modeling, we first flagged any trait-trial for which there was not at least one genetic model with significant $\left(p_{LRT_{\text{null}}}<0.05\right)$ and removed them. Next, among the remaining trials, we removed any that did not have at least one of the non-IID (genomic) models significant (again $p_{LRT_{\text{null}}}<0.05$). The test $LRT_{\text{null}}$ only indicates that the two models being compared are different, not which one is actually better. Therefore, among the remaining trials, we used AIC to determine in which case any of the genomic models were at least as good as the IID model. If $AIC_{\text{IID}}$ was ≥2 units smaller than $AIC_{\text{genomic}},$ we considered the nongenomic model to be better fitting than the genomic one, and subsequently removed those trials.

We used the individual results from per-trial analyses, as described above, to identify and filter out data with very low genetic signal (Table S6). Having curated our dataset as described above, we combined data across trials (within institutes) to achieve larger sample sizes and more replications per clone.

For each Trait-Institute data set, we fit the genetic models ALL, PARTITIONED and non-IDM described above for per-trial analysis. Since the grouped datasets have multiple locations, years, and replications, we added *i.i.d*. random effects for location-year-trial (*LocYrTrial*: trial nested in location-year) and location-year-trial-rep (*LocYrTrialRep*: replication nested in trials).

In addition to $LRT_{\text{null}}$ as described above, we added a test of the significance of the introgression variance $\left(LRT_{\text{partition}}\right)$ by comparing the partitioned model to the non-IDM-only (IDM-null) model.

#### Random partitions:

There were 38K IDMs. Any partition of the genome with this number of SNPs is likely to explain a significant portion of the genetic variance based on that fact alone. We compared the variance explained by the partition according to IDM status to three random sets of SNPs of the same sample size (Tables S7 andS8).

#### LD-adjusted GRMs:

Several previous studies have shown that the primary introgression regions on chromosomes 1 and 4 are characterized by strong, relatively long-range LD (Bredeson *et al.* 2016; Rabbi *et al.* 2017). Excessive (or deficient) tagging of some causal polymorphisms relative to others, for a given trait, is known to bias genetic variance estimates (Speed *et al.* 2012; de los Campos *et al.* 2015). One approach to reduce this bias is to downweight the effect on kinship estimates of SNPs in regions with very high LD, and upweight those in lower LD (Speed *et al.* 2012). We used the software LDAK (version 4.9) to calculate LD-adjustment weights (Table S9), and, subsequently, to construct LD-adjusted GRMs.

We fit the ALL, PARTITIONED, and non-IDM-only models again, this time with the LDAK GRMs in order to observe the impact of LD on the partitioned of variance between IDM and non-IDM.

### GWAS

We conducted two types of GWAS in order to identify QTL attributable to *M. glaziovii* introgressions.

The first GWAS was on the individual SNP markers (both IDM and non-IDM), excluding those with MAF <5%. We ran the mixed-linear model association (*–mlma*) analysis implemented in *gcta* (v1.25.2) (Yang *et al.* 2011). Population structure was accounted by a genetic random effect, $g_{\text{nonIDM}}∼N\left(0,\sigma_{g_{\text{nonIDM}}}^{2}K_{\text{nonIDM}}\right)$. For GWAS with *gcta*, population structure was accounted for by $K_{\text{nonIDM}}$, which, in this case, was constructed using gcta *–make-grm-bin* on non-IDM SNP passing *–maf* 0.01 instead of the rrBLUP::A.mat() version used elsewhere. In the first GWAS, we are able to interpret significant IDM SNPs relative to the *M.g*. diagnostic allele. We further complemented this with a second GWAS, conducted on the introgression-segment dosage matrix (DoseGlaz) based on the 15-IDM windows described above. We conducted this GWAS in R, fitting a mixed-model in which each 15-IDM window was sequentially tested as a fixed-effect, and a random effect with the same covariance $\left(K_{\text{nonIDM}}\right)$ as in the *gcta* analysis. From the estimated marker effects $\hat{\beta }$ and their corresponding SE $se_{\hat{\beta }}^{2}$, we calculated a Wald test statistic:

$$WaldStat={\hat{\beta }}^{2}/se_{\hat{\beta }}^{2}$$

We obtained a *P*-value by computing the probability of observed Wald-statistic under the upper tail of a $\chi^{2}$-distribution with one degree of freedom.

As phenotypic responses for both GWAS, we supplied BLUPs for each clone. Using the *lmer* function in the *lme4* R package, we fit an IID genetic model $\left(g_{IID}\right)$ as described for the per-trial analyses. We analyzed the same curated data and modeled the same design-related random effects (*LocYrTrial* and *LocYrTrialRep*) as in the multi-trial analysis.

### Genomic prediction

We measured the importance of introgression regions for breeding value prediction with fivefold cross-validation. We fit the nonpartitioned (ALL), PARTITIONED, and IDMnull models again using *mmer* in *sommer*. We also fit 15 randomly selected partitions of $n_{\text{nonIDM}}$ including the three using in the multi-trial analysis. Because we used a two-stage genomic prediction approach for cross-validation, computation was much faster, making it possible for us to test more random partitions. The first stage is fitting the models described above for use as response data in GWAS. The second stage is the genomic prediction step, but we first deregressed the BLUPs used for GWAS and weight error variances according to a nonlinear function of the reliability $\left(r^{2}\right)$ and the heritability $\left(h^{2}\right)$. The procedure is described in multiple previous publications (Wolfe *et al.* 2016a,b, 2017) and is based on that described in Garrick *et al.* (2009).

The cross-validation was set-up such that each of the trait-institute data set were divided up into 10 different random partitions of five approximately equal parts. For each model on each fivefold partition of the data, five predictions were made, in which four-fifths of the clones’ phenotypes were included, and one-fifth were left out as a test-set. Prediction accuracy was measured as the correlation among the test-set individuals of their BLUP (as used in GWAS) and their GEBV. For the PARTITIONED model, accuracy was measured for the total GEBV (IDM+non-IDM BLUPs).

### Field plot records of GS progeny

We downloaded records from http://www.cassavabase.org of the number of field plots planted as of 22 January 2019 for each of the GS progeny (Table S2). We reserve phenotypic records for these field plots for a future study.

### Deleterious mutations in introgression regions

We extracted the dosage of deleterious alleles at 9779 of the 22,495 putative deleterious mutations identified by Ramu *et al.* (2017) from a dataset consisting of the LG, GG, and C1, where 5.37 million HapMapII SNPs were imputed. GBS data for the LG, GG, and C1 were imputed in a single step using IMPUTE2 (Howie *et al.* 2009), with the HapMapII serving as a reference panel. IMPUTE2 parameters were set to values similar to those previously used in Lozano *et al.* (2017). Briefly, the number of haplotypes used as a custom reference panel was set to 400, the imputation window was set to 5 Mb and the genetic position for each of the HapMapII markers were interpolated from the composite map published by the International Cassava Genetic Map Consortium (ICGMC) (2014).

### Data availability

The authors state that all data necessary for confirming the conclusions presented in the article are represented fully within the article. Raw unimputed and imputed genotype datasets, downstream analytical results, and high resolution maps of introgressions are publically available on the Cassavabase FTP: ftp://ftp.cassavabase.org/manuscripts/Wolfe_et_al_2019/. Supplemental Figures and Tables files are available at figshare: https://doi.org/10.25386/genetics.9897749.

## Results

### Introgression-associated population structure

In order to detect introgressed *M. glaziovii* genome segments in cultivated cassava samples, we defined IDMs across the genome. When we computed genetic distances from *M. glaziovii*, in order to determine the panel of nonintrogressed *M. esculenta*, we observed elevated variability among HapMapII clones in their genetic distance from *M. glaziovii* on chromosome 1 from Mb 25 to the end of the chromosome (Figure S2). This area corresponds to a region shown previously to be segregating for *M. glaziovii* introgressions (Bredeson *et al.* 2016). Ultimately, we considered only the 120,990 sites intersecting between the HapMapII and the broader GBS dataset, we intended to analyze. From this set, we identified 38,000 IDMs that were either fixed for opposite alleles (*N* = 20,681) or fixed in the *M. esculenta* reference panel, but polymorphic among the *M. glaziovii* (*N* = 17,319). At each IDM locus, therefore, we could identify an allele that was putatively derived from *M. glaziovii* and would be diagnostic of introgression if found in a cultivated cassava genome (Table S1). The IDMs were distributed similarly across the genome compared to the rest of the markers in our dataset (Figure S3).

**Figure 1:**
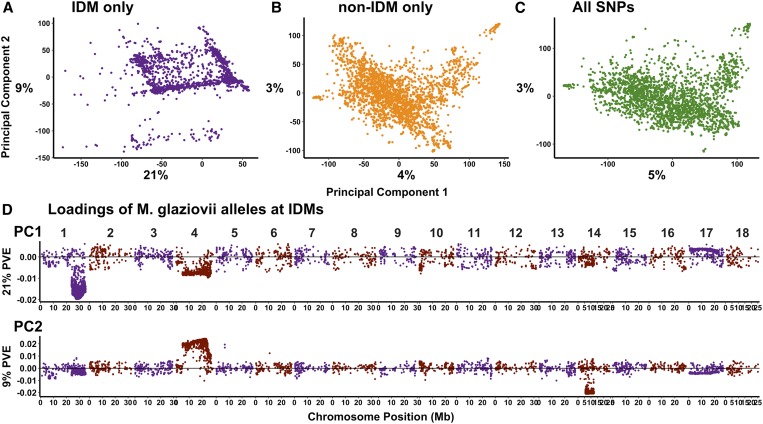
Introgression regions capture distinct population structure. Shown are scatterplots of the PC scores for PC1 and PC2 from three PCAs using three sets of markers: IDM only [(A) excludes “tag” –IDM], non-IDM only (B) and all together [(C) includes “tag” –IDM]. (D) The loadings or eigenvector coefficients from the IDM-only PCA are shown plotted against their genomic coordinates for the first two principal components (vertical panels).

PCA on the IDM markers revealed a pattern of relatedness in introgression regions (Figure 1A) that is distinct from both the rest-of-the-genome (non-IDM only, Figure 1B) and the overall (all SNPs, Figure 1C) analyses. We encoded the dosages for the IDMs to count the *M. glaziovii* diagnostic allele. The resulting loadings (eigenvector coefficients) for markers on PC1 (21% variance explained) are strongest for IDMs on the last 10 Mb of chr. 1, while the strongest loadings on PC2 (9%) are at IDMs spanning the majority of chr. 4 (Figure 1D).

#### Introgression frequency divergence among populations:

**Figure 2:**
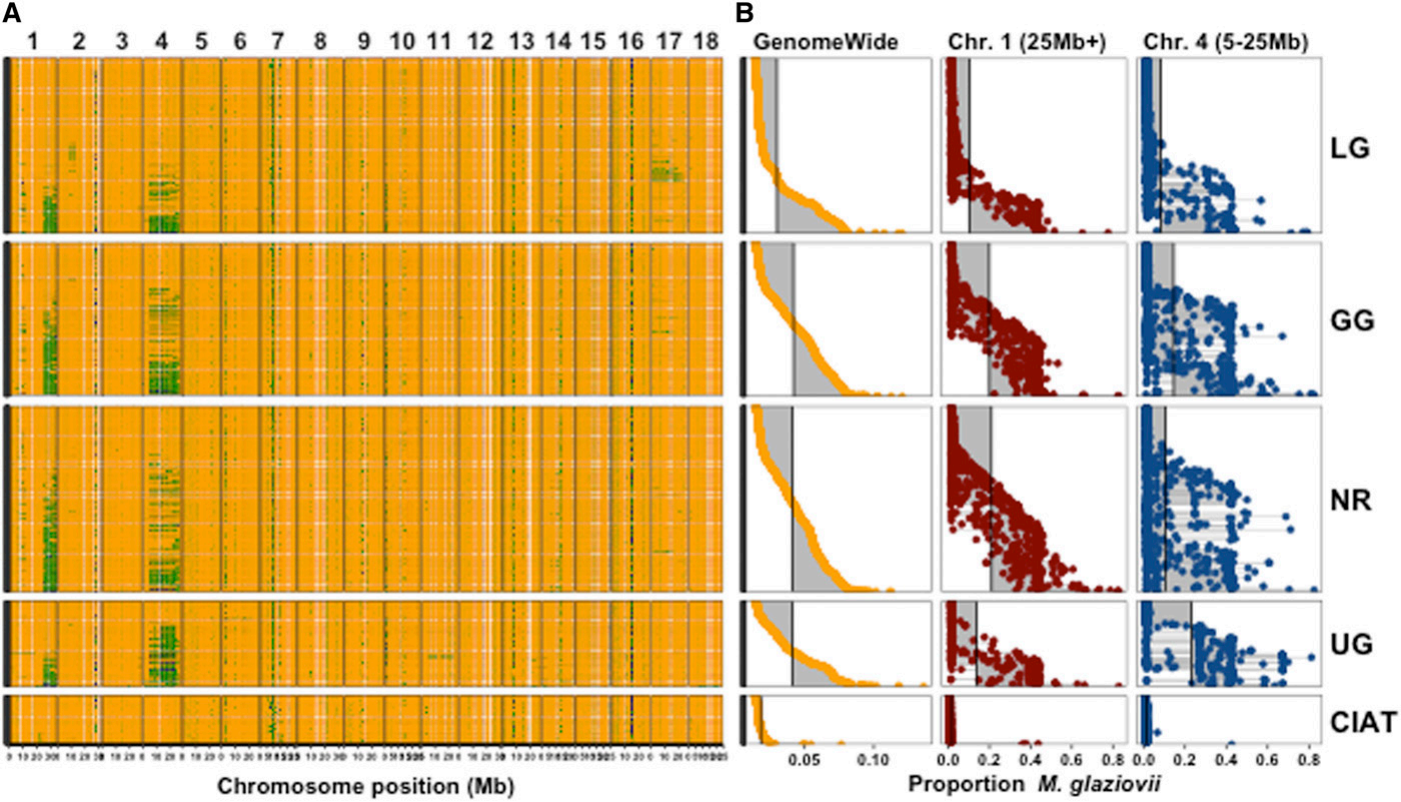
Comparison of introgression among populations. The mean *M. glaziovii* allele dosage at IDMs in 250 kb windows across the genome is depicted on the left (A). Physical position on each chromosome is depicted in megabases (Mb) along the *x*-axis. Colors range from orange (0 M.g. alleles), to green (1 M.g. allele), to dark blue (2 M.g. alleles). The per individual proportion of *M. glaziovii* alleles at IDM is summarized on the right (B). Proportions were calculated as the sum (per clone) of the dosages at IDMs divided by two times the number of IDMs. The proportions in (B) were computed either with all IDMs (GenomeWide, left column), at IDMs on chr. 1 >25 Mb (middle column), at IDMs on chr. 4 from 5 to 25 Mb (right column). The populations can be compared in (B) by looking down the columns and using the vertical lines, which represent the mean values for that group and region, as a visual aid. For both (A and B) each row (*y*-axis) is an individual cassava clone and the vertical panels represent five populations: IITA local germplasm (LG), IITA Genetic Gain (GG), NRCRI (NR), NaCRRI (UG) and the L. American collection (CIAT). Rows (clones) are aligned across (A and B) and sorted within population based on the genome-wide proportion *M. glaziovii* [left column of (B)].

The largest introgressions detected were apparently contiguous segments of chr. 1 ∼25 Mb to the end (∼10 Mb total) and chr. 4 from 5 to 25 Mb (Figure 2A). The genome-wide proportion of *M. glaziovii* alleles per clone ranged from 1.3 to 13.6% (mean 3.8%) among the African breeding germplasm as a whole (GG+LG+NR+UG; Figure 2B, Tables S2, and S3). On a genome-wide basis, there are no large differences among populations in the mean frequency of introgressed alleles. The breeding populations GG (4.2%), NR, and UG (4.1%), have similar levels of introgression, while the L. American collection was the least introgressed (1.8%) and the local germplasm (LG, 3%) was intermediate. We note that in the CIAT collection, the Brazilian accession BRA534 appears to be an outlier, with 34% *M. glaziovii* alleles. We excluded BRA534 when comparing CIAT to other populations.

When we isolate the introgressions on chrs. 1 and 4, which appear to be the same as previously identified by Bredeson *et al.* (2016), we observe more striking differences (Figure 2A). The frequency of the chr. 1 segment was, on average, greater in the W. African breeding germplasm GG (0.2) and NR (0.21) than in the E. African population UG (0.14). In contrast, the introgression on chr. 4 was more common in UG (0.23) compared to GG (0.15) or NR (0.11). Samples from the IITA local germplasm collection (LG) were less likely to contain introgressions on either chrs. 1 (0.10) or 4 (0.08), and the L. American samples from CIAT showed almost no evidence of introgression (<0.02) on both chrs. 1 and 4 (Figure 2B, Tables S2, and S3).

### Ongoing selection for M. glaziovii alleles

**Figure 3:**
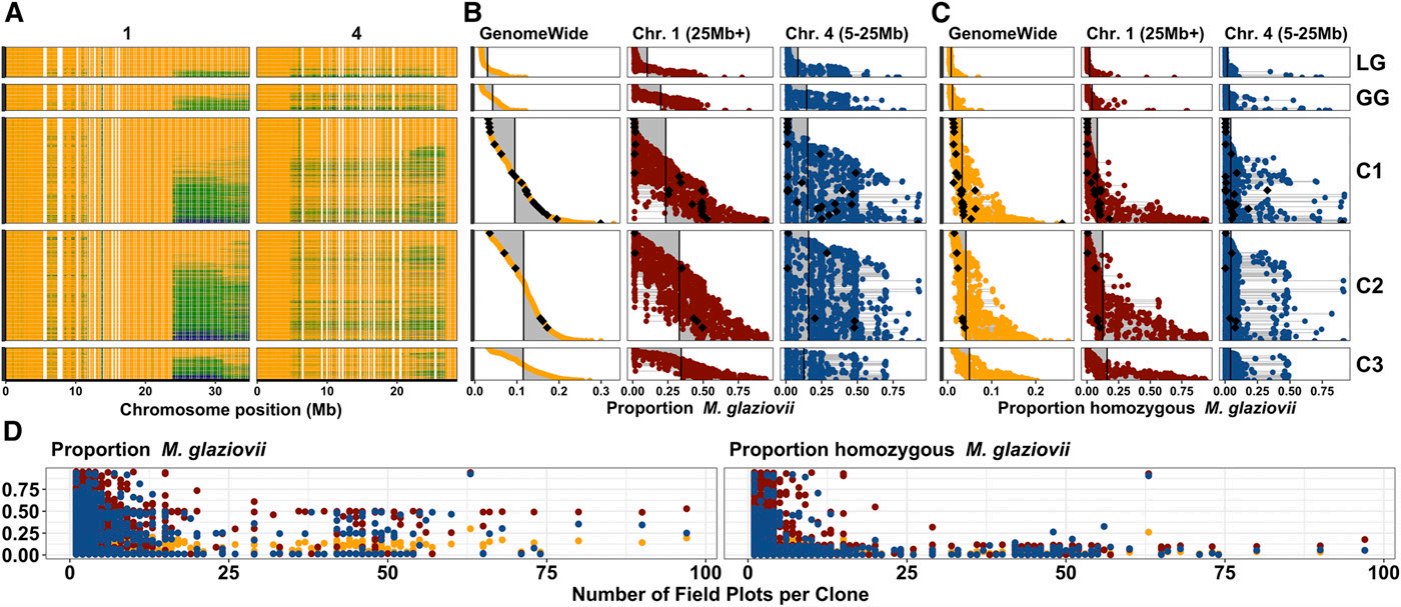
The effects of (genomic) selection on *M. glaziovii* introgressions. The mean *M. glaziovii* allele dosage at IDMs in 250 kb windows on chromosomes 1 and 4 is depicted on the top left (A). Physical position on each chromosome is depicted in megabases (Mb) along the *x*-axis. Colors range from orange (0 M.g. alleles), to green (1 M.g. allele), to dark blue (2 M.g. alleles). The top middle panel (B) shows the per individual proportion and the top right panel (C) shows the rate of homozygosity for *M. glaziovii* alleles at IDM. Proportions for (B) were calculated as the sum (per clone) of the dosages at IDMs divided by two times the number of IDMs. The proportions for (C) were simply the proportion (per clone) out of the total number of IDMs with a dosage equal to two. The proportions in (B and C) were computed either with all IDMs (GenomeWide, left column), at IDMs on chr. 1 >25 Mb (middle column), at IDMs on chr. 4 from 5 to 25 Mb (right column). The populations can be compared in (B and C) by looking down the columns and using the vertical lines, which represent the mean values for that group and region, as a visual aid. For (A–C), each row (y-axis) is an individual cassava clone and the vertical panels represent five populations: IITA Genetic Gain (GG) and three successive generations of genomic selection progeny (C1, C2 and C3), descended originally from GG. Rows (clones) are aligned across (A–C) and sorted within population based on the genome-wide proportion *M. glaziovii* [left column of (B)]. At the bottom, (D) shows how the introgression frequency and homozygosity rate per individual (*y*-axis) for the C1, C2, and C3 relates to the cumulative number of field plots planted (as of January 2019) per clone (*x*-axis). The number of field plots per clone is meant as a proxy representing the level of advancement through variety development stages of the breeding process. For illustrative purposes, we highlight C1, C2, and C3 clones with >50 field plots in (B and C) with black diamonds. For (D) as in (B and C), we break down the proportions in (D) by region and use the same color coding: genome-wide (orange), chr. 1 region (dark red), chr. 4 region (dark blue).

We compared the introgressions detected in local germplasm and landraces of cassava (LG) to IITA improved varieties (GG) and three successive generations of genomic selection progeny (C1, C2, and C3), which descend from parents selected initially from the GG. The most notable changes we observed were on chrs. 1 and 4 (Figure 3A). Genome-wide, the average proportion of *M. glaziovii* alleles per individual increased from 0.03 in LG to 0.042 in GG, and then more than doubled in the GS progeny, with C1 at 0.095, C2, and C3 at 0.12 (Figure 3B, Table S2, and S3). Most of this change was due to increases on chr. 1, which rose from 0.1 in the LG to 0.2 in the GG and maxed out at 0.34 in the C3. In contrast, the chr. 4 region appears to have stayed steady ∼15% from GG through C2 and even slightly decreases from C2 to C3 (Figure 3B, Tables S2, and S3).

Most introgressed LG and GG were heterozygous for *M. glaziovii* haplotypes, with a mean homozygosity rate of only 1% genome-wide (Figure 3C, see also Figure 2A). Genomic selection appears to have steadily increased the homozygosity rate on chr. 1 from 4% in the GG to 16% in C3 (Figure 3, A and C and Tables S2 and S3). The near absence of homozygotes in the elite germplasm (GG) and the gradual increase due to selection that we observed, led us to investigate further.

We hypothesized that postgenotyping performance-based selection and advancement through the variety testing process might exclude homozygous clones. We used the cumulative number of field plots planted (according to http://www.cassavabase.org, January 2019) as a metric of the level of advancement each progeny had attained. We found that while heterozygosity for introgressions was acceptable (Figure 3D, left), homozygous clones were almost completely excluded from later stages (Figure 3D, right). Of the 30 clones with >50 field plots only one of them appeared to be notably homozygous. For that one clone, both chrs. 1 and 4 were nearly completely homozygous (Figure 3D and Table S2).

One potential consequence of increasing the frequency of such a large haplotype and maintaining it in a heterozygous state might be the accumulation of deleterious mutations (Ramu *et al.* 2017). Using a dataset consisting of the LG, GG, and C1 with 5.367 million HapMapII SNPs imputed we were able to genotype 9779 putative deleterious mutations of the 22,495 identified by Ramu *et al.* (2017). From LG to GG, we observed increases in the average per individual genetic load that were larger (34% on chr. 1, 20% on chr. 4) in introgression regions compared to genome-wide (8.7%). Similarly, from GG to C1, genetic load increased, less than between LG and GG, but more in introgression regions (9% for chr. 1 and 4.9% on chr. 4) than genome-wide (2.5%). There was nearly no mean difference between LG and GG in terms of homozygous genetic load. However, there was an increase from GG to C1 and it was also larger in the introgression regions (59% on chr. 1, 15% on chr. 4) than genome-wide (10%) (Tables S2 and S3).

### Local admixture as confirmation of detected introgressions in HapMapII

We also used HAPMIX (Price *et al.* 2009), a haplotype-based method for local ancestry inference, to detect *M. glaziovii* introgressions in phased WGS HapMapII samples. We found that the HAPMIX and IDM-based methods largely agree (Figure S4). Although, we note that *M. glaziovii* segments on Chr. 1 tend to appear smaller in the HAPMIX results.

### Heritability accounted for by introgressions

We quantified the proportion of the total genetic variance that is explainable by introgressions segregating in modern cassava germplasm, for nine traits. We compiled data from 68 field trials (42 IITA, 5 NaCRRI, 21 NRCRI) conducted on 2742 genotyped clones in our study populations (Table S5). To these data, we fit linear mixed-models with two random-genetic effects, kinship measured using IDM markers and kinship by non-IDM markers. The estimated genetic variances partitioned the heritability into two components: one due to introgression regions $\left(h_{IDM}^{2}\right)$ and another for the rest-of-the-genome $\left(h_{nonIDM}^{2}\right)$. Before fitting these models, we performed two major preliminary analyses.

### LD between introgressed regions and the rest of the genome

We first investigated the amount of LD between introgressed and nonintrogressed regions. Using the procedure described in the methods, we reclassified 1413 SNPs, primarily located on chr. 1 and 4 (Figure S7), that were more similar in the kinship they measured to the IDM than to the non-IDM (Figures S5–S7, Tables S1, and S4). Redesignating these SNPs as tag-IDM reduced the correlation of IDM and non-IDM kinships from 0.37 to 0.30. We therefore included tag-IDM in the IDM kinship matrices used in all subsequent analyses.

#### Per-trial analyses:

The second preliminary analysis we did was to analyze each trial separately, in part to check the quality of the data before combining into a larger, multi-trial analysis. Based on a likelihood ratio test, we chose to remove 31 trait-trials that did not show evidence of significant genetic variance $\left(p_{LRT_{null}}>0.05\right)$. We removed an additional six trait-trials without any significant genetic variance from marker-estimated covariances. Lastly, 53 more trait-trials were excluded because, based upon the AIC) the genomic model fit the data worse than the IID model (Tables S6 and S10).

#### Multi-trial analyses:

**Figure 4:**
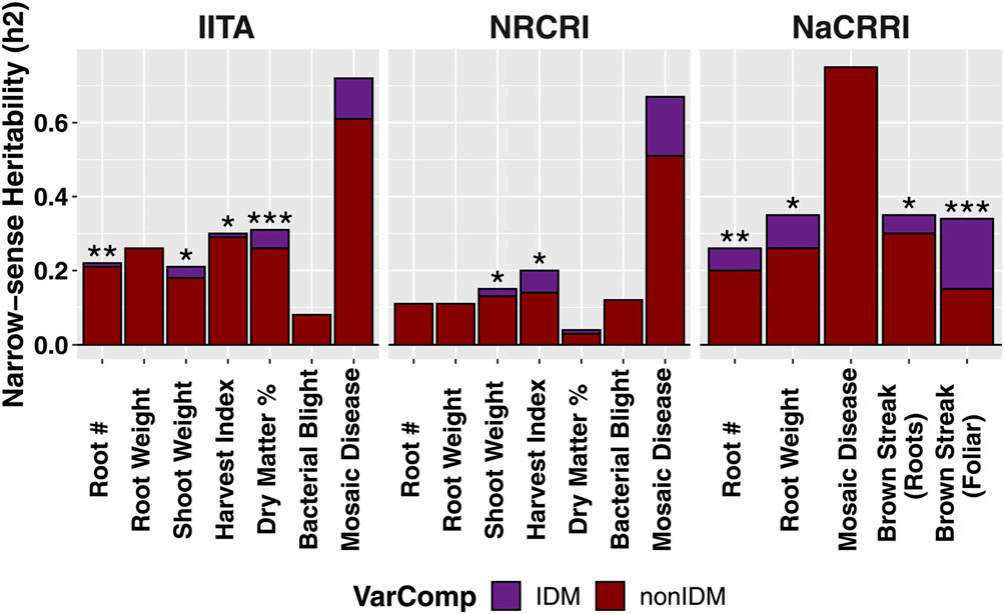
Heritability accounted for by introgressions. The heritability (*y*-axis) of introgression regions for each trait (*x*-axis) measured in each breeding program (horizontal panels). Heritability was estimated from partitioned genomic mixed-models and the portion of heritability attributable to introgression regions (purple) *vs.* the rest of the genome (dark red) is shown. Stars atop bars represent the level of significance in a likelihood ratio test for the significance of the introgression-component (***P < 0.0001, **P < 0.001, *P < 0.05).

We combined the remaining trials for each trait (within Institute) to achieve large overall sample sizes (max per Institute: 25924 IITA, 2881 NaCRRI, 6641 NRCRI) and increase the average number of replications per clone (max per Institute: 16.76 IITA, 6.89 NaCRRI, 8.11 NRCRI; Table S11). We fit the three models for each trait and analyzed each breeding program’s data separately. Out of 19 Trait-Institute analyses, 10 had significant genetic variance from introgressions. In fact, introgression regions appear important for every trait *except* cassava bacterial blight and mosaic disease severity. In all of these cases, the PARTITIONED model had an AIC >2 units smaller than the nonpartitioned one. The proportion of the heritability accounted for by significant introgressions was as high as 56% (mean 20%, median 15%, min 3%; Figure 4, Tables S12, and S13).

#### Comparison to random samples:

One-third of the SNPs in our study were classified as IDMs (including tag-IDMs). We compared the variance explained by our IDM-defined partition, to three random genome partitions of the same size (Table S7). For the random samples, the correlation of GRMs was >0.99, but was only 0.30 for the IDM-defined partition (Table S8). The IDM-defined partition explained an average of 20% of the total genetic variance, in comparison to 37% for the random partitions, which is closer to proportional with the total number of markers (Figure S8, Table S12–S14).

Most of the cases with significant $\sigma_{\text{IDM}}^{2}$ did not have significant variance associated with the random samples of equivalent size. In contrast, all three random samples had significant variance for MCMDS in the IITA dataset, while the equivalent IDM-defined variance was insignificant. For the most part, AIC values indicate the IDM-defined partitions fit at least as well, if not better, than the random ones. For only two cases did a random sample appear to fit better than IDM-defined (MCMDS IITA Sample 1, RTNO IITA, Sample 2). In the NaCRRI dataset, IDM-defined partitions fit better than all three random samples for MCMDS and MCBSDS. For the better fitting (compared to random) NaCRRI MCMDS analysis, the variance from the IDM regions was actually zero.

#### Importance of LD:

We know from previous studies in cassava, and confirm here (Figure S9 and Table S21), that the introgression regions on chromosomes 1 and 4 are characterized by strong, relatively long-range LD (Bredeson *et al.* 2016; Rabbi *et al.* 2017). We computed the cumulative genetic size in centimorgans in 1-Mb windows along each chromosome (Table S21). We found that recombination was 14 and 71% less than the rest of the genome in the introgressions on chrs. 1 (25 Mb+) and 4 (5–25 Mb), respectively. We used the LDAK method (Speed *et al.* 2012) to weight SNPs contributions to kinship matrices (GRMs) in order to correct for variability in tagging of causal mutations due to LD (Speed *et al.* 2012; de los Campos *et al.* 2015) (Figures S10, S11, and Table S9). The key result of LD adjustment was a mean decrease of 7.4% of the proportion $h_{\text{IDM}}^{2}/h_{\text{Total}}^{2}$ (max decrease −41.6% for CBSDRS, max increase 6.6% for RTNO), among cases where at least one of the models had significant $LRT_{\text{IDM}}$ (Figures S12, S13, and Table S17). We noted that the off-diagonals of the LDAK adjusted IDM and non-IDM GRMs were more correlated to each other (0.65) than the unadjusted pair (0.3; Table S16). The IDM matrix was altered most by LDAK adjustment, with off-diagonal correlation to the unadjusted IDM matrix of 0.45 compared to 0.89 for the non-IDMs (Table S15). In all, 12 $\sigma_{IDM}^{2}$ were significant, either before, after, or both before and after LD-adjustment. Of these 12, there were two where LD-adjustment made the $LRT_{\text{IDM}}$ significant and three where it became insignificant.

### M. glaziovii-associated QTL

We identified QTL attributable to *M. glaziovii* alleles using mixed-linear model GWAS on two types of predictors. The first GWAS was on the SNP markers themselves, and the second was on the mean dosage of *M. glaziovii* alleles in 250-kb windows (*DoseGlaz*), described in more detail in *Methods*. There were Bonferroni-significant IDM and/or *DoseGlaz* for all traits except bacterial blight (MCBBS) (Figure 5, Figures S14, and S16–S25).

**Figure 5:**
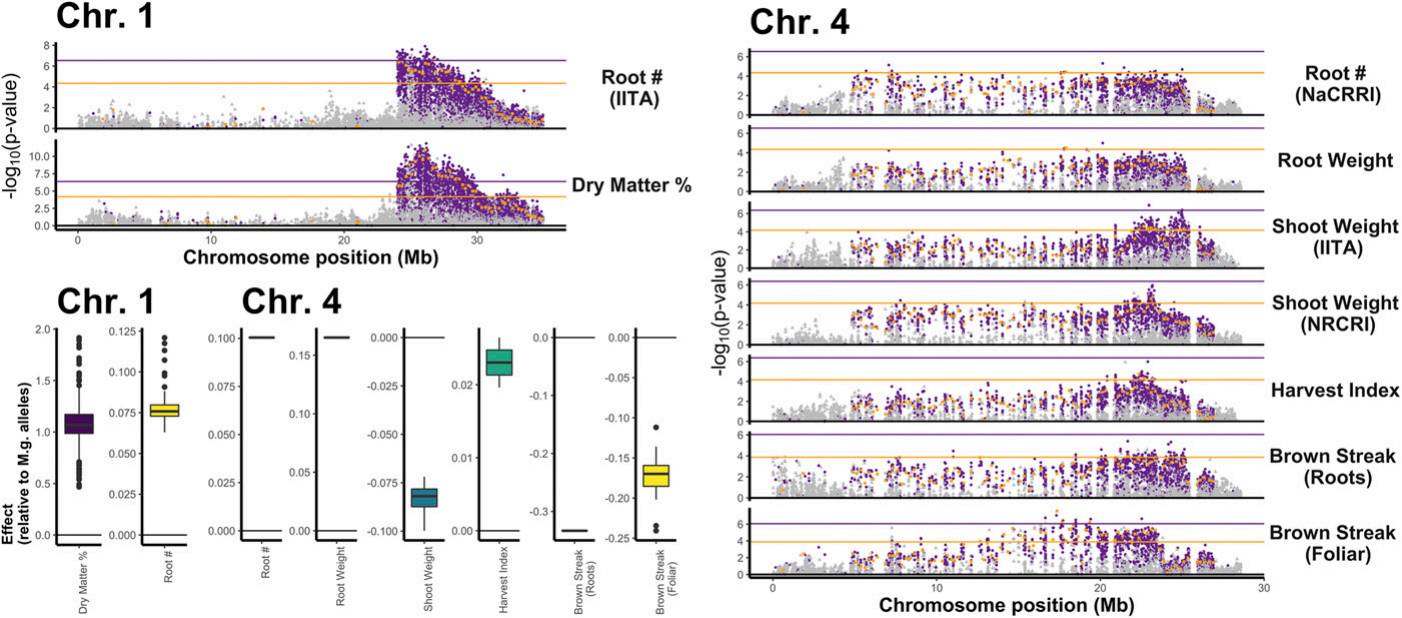
Significant introgression-trait associations. Manhattan plots summarizing genome-wide associations for traits with significant introgression-trait associates on chromosomes 1 and 4. Two mixed-linear model association analyses are shown, overlaid. In the first, GWAS was conducted on IDM (purple) and non-IDM (gray) SNP markers. For the second, GWAS 250 kb window-based mean *M. glaziovii* allele dosages at IDMs (*DoseGlaz*; orange squares). The horizontal lines represent the Bonferroni-significance threshold for the *DoseGlaz* (orange line) and SNP GWAS (purple line). In the bottom left quadrant is a boxplot of all Bonferroni-significant marker-effects pooled by trait and chromosome.

On chr. 1 between 24.0 and 31.9 Mb, significant IDM and *DoseGlaz* were detected for DM (mean effect of *M. glaziovii* alleles in percent DM: 1.05 IDM, 1.49 *DoseGlaz*) and RTNO [mean effect in ln(kg/plot): 0.08 IDM, 0.09 *DoseGlaz*] (Table S18). For MCBSDS, the Chr. 4 QTL includes *DoseGlaz* and IDM, covering most of the introgression region, from 12.6 to 23.4 Mb. For SHTWT and HI however, the region spanned only from 22.35 to 25.1 Mb and there was a single significant marker for RTNO and RTWT nearby at 17.9 Mb. Effects on chr. 4 of *M. glaziovii* alleles for brown streak disease appear protective [mean effect on disease severity (1–5) score: −0.17 MCBSDS, −0.33 CBSDRS]. For SHTWT [units: ln(kg/plot)] and HI (units: proportion 0–1) mean *M. glaziovii* effects were −0.085 and 0.023, respectively. In addition, there was one *DoseGlaz* significant for MCBSDS on chr. 5 and one on chr. 12. The sig. *DoseGlaz* on chr. 12 was estimated to *increase* disease susceptibility with an effect-size of 1.22 (trait scale 1–5). Note that RTNO and SHTWT effects are on the natural log scale.

### Impact of introgressions on genomic prediction

GS is becoming an important part of modern cassava breeding (Ly *et al.* 2013; Wolfe *et al.* 2017). We investigated the impact of introgression regions on genomic prediction accuracy, which is directly proportional to their contribution to breeding gains during GS, all other things being equal. We did 10 replications of fivefold cross-validation for each trait-institute dataset. We tested five prediction models: nonpartitioned (ALL markers), genome-partitioned, and IDM-null models. For the “genome-partitioned” and “IDM-null” models, we divided markers into two kinship matrices, either randomly or based on whether a SNP was an IDM or not.

**Figure 6:**
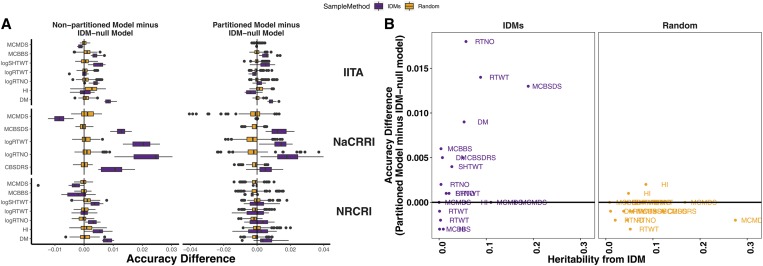
The importance of introgressions for genomic prediction. (A) The difference in prediction accuracy between a model with *vs.* without the introgression regions is expressed in horizontal boxplots. Ten replications of fivefold cross-validation was conducted for each Trait-Institute combination. For each trait-institute dataset, we used the same 10 random partitions of training-test for each model tested. Two measures are shown on the *x*-axis: the total accuracy of the partitioned model minus the IDM-null model [(A), left], the accuracy of the nonpartitioned model minus the IDM null model [(A), right]. Two methods of partitioning the genome were compared: the IDM-based partition (purple boxplots), and 15 different random partitions, pooled in the (orange) boxplots. (B) The mean difference in prediction accuracy between the partitioned model and the IDM-null is plotted (*y*-axis) against the introgression-associated heritability (*x*-axis) from the multi-trial analyses. Results are shown for the IDM-based partition of the genome (purple, left panel) and three tested random partitions (orange, right panel).

The accuracy of partitioned models was almost identical to the nonpartitioned model for both the IDM-based and the random genome-partitions. However, removing the IDM-based component from the model tended to reduce accuracy, especially in the NaCRRI data, on average 0.004 (max 0.04) relative to the PARTITIONED and 0.005 (max 0.03) relative to the ALL models (Figure 6A). These comparisons provide a means to measure the importance of introgression regions in ongoing GS. In contrast to the IDM-based genome partition, removing the equivalent random components decreased accuracy an average 0.001 (compared to ALL) and −0.001 (compared to PARTITIONED) (Figure S15, Tables S19, and S20).

Finally, we observed that the size of the impact on prediction accuracy (measured from comparing ALL and IDMnull models) scaled with the $h_{\text{IDM}}^{2}$ with a correlation of 0.41 for the IDM-based genome partition and −0.09 for the random partition (Figure 6B).

## Discussion

### Beneficial effects of introgressed alleles are consistent with divergence in their frequencies across the African continent

The original impetus for interspecific hybridization at Amani (circa 1930s) was to combat CMD (Nichols 1947; Jennings 1957; Hahn *et al.* 1980b; Legg and Thresh 2000; Jennings and Iglesias 2001). We observed consistent and beneficial *M. glaziovii* allelic effects; however, we found neither a beneficial effect nor a significant genetic variance for CMD. In a previous article, focused on GWAS for CMD, we noted an absence of major effect QTL other than CMD2, a dominant, possibly multi-allelic locus (Wolfe *et al.* 2016b). We verify here that the protection derived from the CMD2 locus did not arise from introgression, as the only associated GWAS result on Chr. 12 indicated the *M. glaziovii*-allele increased susceptibility (Figure S16 and Table S18). Introgression-derived CMD resistance has previously been suggested to be weak (relative to CMD2), “recessive” and “polygenic” (Fregene *et al.* 2000; Lokko *et al.* 2006); our results seem to be in agreement with this assessment.

Introgression alleles we did detect at QTL are adaptive and consistent with the population structure we observed (Figure 1A), arising primarily due to segregation of the two very large segments detected on Chr. 1 from 25 Mb to the end and Chr. 4 from 5 to 25 Mb, as well as a segment on Chr. 14 (Figure 1B, Figure 2, and Figure 3). *M. glaziovii* segments are common in African breeding germplasm (Figure 2), less common among African landraces, and nearly absent from Latin American cassava. Dry matter alleles from *M. glaziovii* at a previously identified QTL on chr. 1 [Figure 5; Rabbi *et al.* (2017)] seem to explain the higher frequency of those introgression segments in W. Africa, given the breeding emphasis placed there on that trait as well as yield. The chr. 4 segment, in contrast, is more common in east Africa, which also aligns with the focus there on CBSD resistance breeding (Hillocks and Jennings 2003; Kawuki *et al.* 2016) and the protective *M. glaziovii* alleles there for that disease (Figure 5) (Nzuki *et al.* 2017; Kayondo *et al.* 2018). The breeding focus on DM and yield in W. Africa also explain the differential change over IITA GS cycles in introgression frequency on chr. 1 compared to chr. 4. We note that, in line with a recent study of cross-continent prediction of CBSD resistance (Ozimati *et al.* 2018), the existence in West Africa of the potentially protective chr. 4 segment is promising for the possibility to preventatively breed for CBSD resistance in W. Africa. This leads to the reciprocal suggestion that any beneficial DM alleles being targeted in W. Africa are likely to be present and thus potentially useful in E. Africa.

By comparison of African to Latin America clones, we believe our evidence supports the origin of the chr. 1 and 4 *M. glaziovii* introgressions African, in line with historical and recent genomic evidence (Bredeson *et al.* 2016). We do note five CIAT clones with signatures of introgression; one is BRA534, which at 34% *M. glaziovii* genome-wide, likely has recent (non-African) wild ancestors; the four others were heterozygous for the same segments on chrs. 1 and 4 that the African germplasm have. To date, we have not been able to trace the pedigree or otherwise ascertain the origin of these clones.

### Inbreeding depression due to linkage drag accumulating genetic load in introgression regions may explain homozygote deficit among landraces and elite cultivars

The suppression of recombination, often due to structural variants like inversions, is often a consequence of hybridization between crops and their wild relatives (Rieseberg *et al.* 2000; Fijarczyk and Babik 2015; Llaurens *et al.* 2017). Though we do not know whether an inversion or other structural variant underlies *M. glaziovii* introgressions in cassava, we estimated that recombination is reduced in the introgressed regions on chr. 1 and 4 by 14 and 71% respectively, compared to the rest of the genome [Figures S6, S9, and Table S21; Bredeson *et al.* (2016); Rabbi *et al.* (2017)]. Further, adjusting for LD using LDAK almost uniformly reduced the heritability accounted for by introgressions (Figures S12 and S13). Also, though the introgressions were clearly important for genomic prediction, their overall effect on accuracy was small (Figure 6 and Figure S15). This suggests that while introgressions are clearly still important, having uniformly beneficial effects at QTL (Figure 5) and nearly doubling in frequency during three cycles of GS (Figure 3), their physical size is disproportionate to their true economic value.

One theoretical prediction about introgressed alleles under selection with suppressed recombination is that they can result in the accumulation of genetic load due to linkage drag (Haldane 1957; Fijarczyk and Babik 2015). This is especially a concern for vegetatively propagated noninbred crops like cassava (Ramu *et al.* 2017). We observed that putatively deleterious alleles in introgression regions accumulated relatively faster (both LG to GG and GG to C1) compared to the rest of the genome. We further observed balancing selection in the form of an *M. glaziovii* homozygote deficit from variety trials.

In clonally propagated crops, selection for advancement during variety trials is using total genetic merit rather than breeding value based on performance in a series of field trials with progressively more replicates, locations, and increasing plot size. The GS progeny that we analyzed (and thus the sample in which we observed an initial increase in *M. glaziovii* homozygosity) represent those that successfully germinated and were vigorous enough early on to warrant genotyping. We discovered that *M. glaziovii* homozygotes were excluded from later stage field trials early in the process (Figure 3D). This indicates there may be phenotypically expressed negative effects of these introgressions, which may be related to the accumulation of homozygous deleterious mutations we observed. Linkage drag in adaptive introgression regions has been proposed to explain balancing polymorphism regions in cases including human-Neanderthal hybridization (Harris and Nielsen 2016) and wing mimicry in *Heliconius* butterflies (Joron *et al.* 2011; Le Poul *et al.* 2014).

Introgression-associated inbreeding depression is thus a critical topic for future investigation. At present, cassava breeders are maintaining introgression heterozygosity at great cost, through a multi-stage selection process. First, favored crosses between heterozygous parents generates many unsuitable offspring, which are homozygous for introgressions and suffer some as yet unquantified inbreeding depression. Subsequently, field evaluations are required to identify and purge these individuals. We propose that targeted recombination of introgression segments would increase the rate of gain and sustainability of cassava breeding by allowing simultaneous fixation of beneficial alleles and purging of genetic load.

Taken together, our results point to the continued importance of wild alleles in cassava, one of the most important staple foods in the developing world, and a model for other clonally propagated root and tuber crops. We present an example of both the benefits and consequences of historical introgression for modern crop breeding. Our methods and the breeding implications we highlight will therefore provide a valuable example for other crops.
